# Electrochemical immunosensor for detection of *Plasmodium vivax* lactate dehydrogenase

**DOI:** 10.1590/0074-02760220085

**Published:** 2022-08-26

**Authors:** Ariamna María Dip Gandarilla, Juliane Correa Glória, Yonny Romaguera Barcelay, Luís André Morais Mariuba, Walter Ricardo Brito

**Affiliations:** 1 Universidade Federal do Amazonas, Central Analítica Multidisciplinar, Laboratório de Bioeletrônica e Eletroanalítica, Manaus, AM, Brasil; 2 Universidade Federal do Amazonas, Departamento de Química, Manaus, AM, Brasil; 3 Fundação Oswaldo Cruz-Fiocruz, Instituto Leônidas e Maria Deane, Manaus, AM, Brasil; 4 Universidade Federal do Amazonas, Departamento de Física, Manaus, AM, Brasil

**Keywords:** electrochemical immunosensor, malaria, *Plasmodium vivax* lactate dehydrogenase, IgY antibodies

## Abstract

**BACKGROUND:**

Malaria is a disease that affects many tropical and subtropical countries, including Brazil. The use of tests for malaria detection is one of the fundamental strategies recommended by the World Health Organization for the control and eradication of the disease. The lack of diagnostic tests leads to an increase in transmission and non-reporting cases.

**OBJECTIVES:**

This work described an electrochemical immunosensor for detecting *Plasmodium vivax* lactate dehydrogenase antigen (Ag-*Pv*LDH).

**METHODS:**

The device has developed by immobilising egg yolk IgY antibodies (Ab-*Pv*LDH) on a gold electrode surface using cysteamine as linker. The immunosensor fabrication was followed by differential pulse voltammetry, and contact angle measurements were performed to characterise the modified gold electrode surface.

**FINDINGS:**

The results for Ag-*Pv*LDH determination exhibit a linear response at 10-50 µg mL^-1^ concentration range, with a limit of detection of 455 ng mL^-1^. The excellent selectivity of the device was confirmed.

**MAIN CONCLUSIONS:**

The developed immunosensor showed a good performance, therefore, it can be considered an alternative test to detect malaria caused by *P. vivax*.

Malaria infection is caused by *Plasmodium* sp parasites and transmitted to humans by *Anopheles* sp mosquitoes. According to World Health Organization (WHO), 241 million cases were registered in 2020, a 6.16% higher when compared to 2019. Most of these cases increase was reported in the African Region. Moreover, were estimated 627,000 deaths by malaria, which represents more than 69,000 compared to 2019.^1^ About two thirds of the additional malaria deaths were due to disruptions in the provision of malaria prevention, diagnosis, and treatment during the pandemic situation caused by Covid-19.^2^ In Brazil, malaria is an endemic disease in the Amazon region, which constitutes a serious public health problem. In 2020, 145,188 cases and 45 deaths were registered, showing an increase of 18.9% in the number of deaths compared to 2019. *Plasmodium vivax* sp is the most common in the Amazon region.^3^

Lactate dehydrogenase (LDH) is a water-soluble enzyme produced by parasites’ sexual and asexual stages. Expressed in high concentrations, it is essential for the anaerobic generation of adenosine triphosphate and catalyses the parasite’s glycolytic pathway. That biomolecule is considered a malaria biomarker and can be detected in biological samples (blood or serum) from infected patients.^4^

Currently, the confirmatory diagnosis for malaria can be performed by different methods such as microscopic, immunological (enzyme-linked immunosorbent assay - ELISA - and rapid diagnostic tests - RDTs), and molecular (nucleic acid amplification tests - NAATs) techniques.^5^ ELISA and NAATs methods are highly sensitive; however, they are time-consuming, require expensive reagents and equipment, specialised laboratories, and skilled personnel.^6^ ^,^ ^7^ On the other hand, RDTs are low cost, have short analysis times, and require unskilled labor, but their sensitivity is lower, and the results may be influenced by storage and execution conditions.^8^ ^,^ ^9^

In the last 20 years, the development of sensing platforms, like electrochemical immunosensors, has increased significantly. Their application has been extended to different areas such as agriculture, food, quality control, environmental and industrial surveillance, and medical applications in the detection of diseases.^10^ ^,^ ^11^ ^,^ ^12^ ^,^ ^13^ The choice of adequate strategies for antibody immobilisation is an important factor to obtain devices with high performance, stability and applicability. This is one reason why many researches are focused on signal amplification, with the aim of increasing the limits of detection (LOD).^14^

While antibodies remain the key recognition element in biosensor development, some limitations are widely known, such as expensive and time-consuming production, and its obtention requires experimentation in animals and needs adequate facilities.^15^ Immunoglobulins of Y type (IgY) from egg yolk have been applied for therapeutic purposes and in the development of immunoassays due to the production and purification processes present some advantages when compared to IgG.^16^ ^,^ ^17^ ^,^ ^18^ ^,^ ^19^

In this work, we presented an electrochemical immunosensor for the detection of Ag-*Pv*LDH. The sensing platform was fabricated by immobilising egg yolk IgY antibodies (Ab-*Pv*LDH) on gold electrodes (GE) surface modified with self-assembled monolayers (SAMs) of cysteamine.

## MATERIALS AND METHODS

*Reagents* - All reagents were of analytical grade, and the aqueous solutions were prepared using purified water from a Milli-Q system. Cysteamine (Cys), glutaraldehyde (Glut), bovine serum albumin (BSA), potassium hexacyanoferrate (III), glucose, glycine, and potassium chloride were purchased from Sigma-Aldrich (USA). Potassium hexacyanoferrate (II) trihydrate, sulfuric acid (98%), and phosphate buffer saline (PBS) of pH 7.00 were acquired from Merck (Germany).

The biomolecules preparation process was performed at Leônidas and Maria Deane Institute (Oswaldo Cruz Foundation - Manaus - Brazil). Ag-*Pv*LDH consisted of a recombinant expression of *P. vivax* LDH in *Escherichia coli* BL21 pLysS (Thermo Fisher Scientific, USA), and the purification was performed using affinity chromatography nickel columns (Qiagen, Germany), following the manufacturer instructions.^20^ 7-week-old Dekalb White laying hens were immunised with peptides coupled to solubilised carbon nanotubes. The antibodies (polyclonal Ab-*Pv*LDH (IgY)) were extracted from chickens’ egg yolks and purified using an AminoLink Coupling Resin (Thermo Scientific, USA), coupled with the antigen (1 - 2 mg of peptide for 1 mL of resin).^21^ Also, Histidine-rich protein 2 of *Plasmodium falciparum* (Ag-*Pf*HRP2) was previously synthesised^22^ and used in the selectivity studies.

*Equipment* - All electrochemical measurements were performed using a PGSTAT128N (Methrohm Autolab, Netherlands), with NOVA software, 2.1.5 version. The electrochemical cell was constituted by a three-electrode system, with a disc GE as the working electrode, Ag/AgCl as reference electrode (sat. KCl), and platinum as the auxiliary electrode. Contact angle (CA) measurements were made using a digital microscope (Dino-Lite plus). 1μL of Milli-Q water was added to the surface of bare GE and modified with cysteamine, and the droplet behavior was observed for 120 seconds at 22 ± 1ºC. The CA values were obtained in triplicate using the Image J software. Besides, an ultrasonic bath (QUIMIS, Q335D model, Brazil) was used for cleaning the GE.

*Immunosensor preparation* - First, the GE (2 mm in diameter) was polished with 0.3 and 0.05 μm alumina slurry, polishing sandpaper (P4000), and cloth pad (Buehler, USA), followed by ultrasonication in Milli-Q water for 3 minutes. Then, an electrochemical cleaning was performed in 0.5 mol L^-1^ H_2_SO_4_ solution, through cyclic voltammetry, with a potential range of 0.0 to 1.5 V (vs. Ag/AgCl, sat. KCl) at a scan rate of 50 mV s^-1^. Afterward, the GE were rinsed with Milli-Q water and dried using a nitrogen gas stream.

For immunosensor fabrication, SAMs were formed on a bare GE surface. The process consisted of immersing the GE in cysteamine solution (50 mmol L^-1^, prepared in 99% ethanol) for 4h at 25ºC. Then, 3 µL of 2.5% glutaraldehyde solution were deposited and kept in a humid chamber for 1 h. In the next step, 3 µL of Ab-*Pv*LDH antibodies (IgY type) (0.2 µg µL^-1^) were deposited on GE and incubated for 4 h at 4ºC in a humid chamber. The surface was blocked with 1% BSA for 1 h in a humid chamber to avoid unspecific antigen-binding during the detection step. Finally, the immunosensor was incubated in Ag-*Pv*LDH solution at different concentrations (10-50 µg mL^1^) for 1 h in a humid chamber. After each modification step, the electrode was gently washed with Milli-Q water and dried at room temperature. The electrical response was recorded during all modification steps by differential pulse voltammetry (DPV), with potential between 0 and 0.45 V, at 25 mV s^-1^ in 5 mmol L^-1^ K_3_[Fe(CN)_6_]/K_4_[Fe(CN)_6_] + 0.1 mol L^-1^ KCl.

## RESULTS AND DISCUSSION

**Fig. 1:**
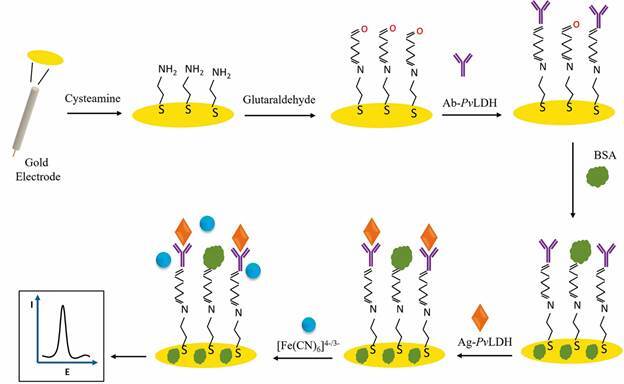
schematic representation of immunosensor fabrication steps and analytical procedure for *Plasmodium vivax* lactate dehydrogenase antigen (Ag-*Pv*LDH) determination.

The manufacturing steps of Ag-*Pv*LDH/BSA/Ab-*Pv*LDH/Glut/Cys/GE are represented in Fig. 1. Firstly, the incubation in cysteamine solution leads to the spontaneous formation onto the GE surface of a monolayer through gold-sulfur interaction. The monolayer is constituted by primary amines, where the terminal amino groups are exposed to the surrounding environment. These groups are modified with glutaraldehyde, which presents two-terminal aldehyde functional groups. One reacts with amines through a nucleophilic addition mechanism, forming imine-like covalent bonds, while the other aldehyde group remains free to react with an amine group of the antibody. The subsequent modification with BSA, blocks the unmodified gold surface and the aldehyde functions that did not interact with the antibodies. Later, Ag-*Pv*LDH was selectively recognised by the antibodies, leading to changes in the K_3_[Fe(CN)_6_]/K_4_[Fe(CN)_6_] electrochemical response, which were recorded by DPV technique.^23^

**Fig. 2:**
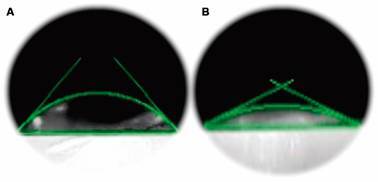
imagens of contact angle for A: bare gold electrodes (GE) and B: GE modified with cysteamine.

The hydrophobicity/hydrophilicity of a surface can be evaluated through CA measurements [hydrophilic (< 90º), hydrophobic (> 90º), or superhydrophobic (> 150º)], where the greater interaction of the liquid with the surface leads to lower CA values.^24^ Fig. 2 shows the CA images for bare GE and modified with cysteamine. The values calculated before and after modification were 45.65º and 18.19º, respectively.

CA measurements confirmed the presence of the SAMs on GE. After SAMs formation, a noticeable decrease in CA values was observed. Similar behavior has been reported by other authors^25^ ^,^ ^26^ and can be explained by terminal amino groups in the cysteamine structure attributing greater hydrophilicity to the surface.

**Fig. 3:**
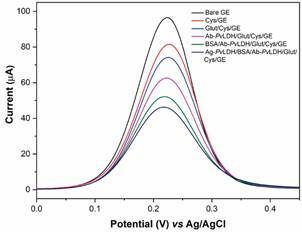
differential pulse voltammetry (DPV) recorded by *Plasmodium vivax* lactate dehydrogenase antigen (Ag-*Pv*LDH)/bovine serum albumin (BSA)/*P. vivax* lactate dehydrogenase antibodies (Ab-*Pv*LDH)/glutaraldehyde (Glut)/Cysteamine (Cys)/gold electrodes (GE) in 5 mmol L^-1^ K_3_[Fe(CN)_6_]/K_4_[Fe(CN)_6_] + 0.1 mol L^-1^ KCl.

*Electrochemical characterisation of the immunosensor* - All modification steps of GE were followed by DPV (Fig. 3). The voltammogram of bare GE recorded the highest values of anodic peak current (Ipa = 96.43 µA). The results obtained after incubation in cysteamine and glutaraldehyde solutions exhibited a decrease in the amperometric response (Ipa = 81.62 µA and Ipa = 74.17 µA, respectively) due to the lower access of the redox probe ([Fe(CN )_6_]^4-/3-^) to the electrode. Besides, at pH = 7.40 the amino groups are quasi-neutral, and the cysteamine monolayer exhibits insulating characteristics.^26^ Several authors have reported that behavior using SAMs formation methodologies in the development of biosensors.^23^ ^,^ ^26^ ^,^ ^27^

After immobilisation of Ab-*Pv*LDH was observed a decrease in current values (Ipa = 62.57 µA) compared to the previous modification step due to the large molecular size of these antibodies (~180 kDa), which blocks the transfer of charge. Then, with the presence of BSA on the surface, a similar behavior was evidenced (Ipa = 52.16), justified by the existence of another protein of large size (~66 kDa). Finally, when the immunosensor was incubated in Ag-*Pv*LDH solution (10 µg mL^-1^), another decrease in Ipa values was recorded (Ipa = 46.30) due to antibody-antigen interaction.

**Fig. 4:**
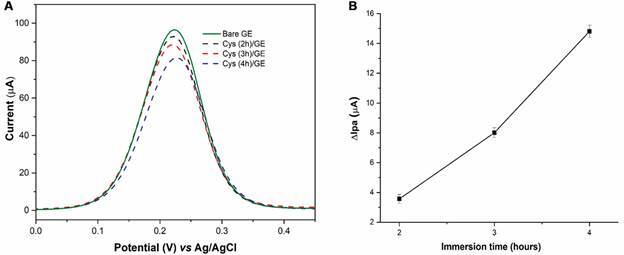
optimisation of immersion time in cysteamine solution. A: differential pulse voltammetry (DPV) in 5 mmol L^-1^ K_3_([Fe(CN)_6_(]/K_4_([Fe(CN)_6_(] + 0.1 mol L^-1^ KCl and B: Relation ∆Ipa versus immersion time.

The formation of SAMs on GE surface was optimised, and three immersion times (2, 3, and 4 h) in cysteamine solution (50 mmol L^-1^) were tested. The Ipa values show a decrease with the increase of the immersion time (Fig. 4A). A ∆Ipa (Ipa _before cysteamine_ - Ipa _after cysteamine_) was calculated (Fig. 4B) and adopted 4 h as the optimum time for the SAMs formation.

Other parameters such as optimum antibody concentration was used according to Figueiredo et al.,^28^ who developed with excellent results an immunosensor through immobilisation of egg yolk antibodies on gold electrodes for dengue detection.

**Fig. 5:**
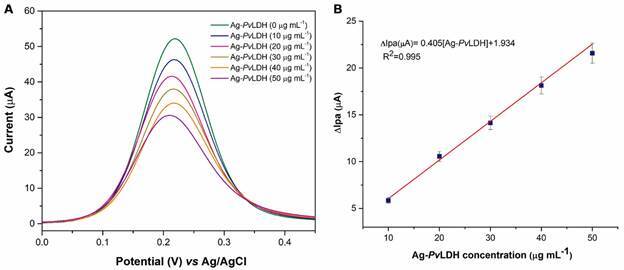
A: differential pulse voltammetry (DPV) recorded in 5 mmol L^-1^ K_3_[Fe(CN)_6_]/K_4_[Fe(CN)_6_] + 0.1 mol L^-1^ KCl with the *Plasmodium vivax* lactate dehydrogenase antigen (Ag-*Pv*LDH)/bovine serum albumin (BSA)/*P. vivax* lactate dehydrogenase antibodies (Ab-*Pv*LDH)/glutaraldehyde (Glut)/Cysteamine (Cys)/gold electrodes (GE) immunosensor upon additions of Ag-*Pv*LDH at different concentrations and B: Calibration curve (∆Ipa versus Ag-*Pv*LDH concentration).

*Determination of Ag-PvLDH* - The analytical performance of the immunosensor was examined with Ag-*Pv*LDH standard solutions (n = 5) at different concentrations: 10, 20, 30, 40, and 50 µg mL^-1^ in 0.1 mol L^-1^ PBS of pH 7.00. The results showed a decrease in Ipa values with increasing antigen concentration (Fig. 5A), produced by the electrode passivation at higher concentrations. A linear relationship was observed from 10 to 50 µg mL^-1^ Ag-*Pv*LDH, with a LOD of 455 ng mL^-1^.

The ∆Ipa was calculated from the Ipa values before and after incubation in Ag-*Pv*LDH solution (∆Ipa = Ipa _*(before Ag-PvLDH)*_ - Ipa _*(after Ag-PvLDH)*_ ). The calibration curve (Fig. 5B) was obtained by plotting of ∆Ipa (µA) versus Ag-*Pv*LDH concentration (µg mL^-1^) and was defined by ∆Ipa (µA) = 0.405[Ag-*Pv*LDH] + 1.934, with R^2^ = 0.995 (Fig. 5B).

**Fig. 6:**
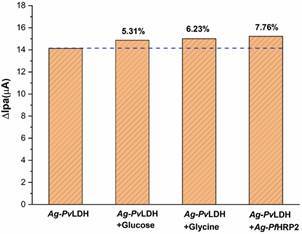
differential pulse voltammetry (DPV) in 5 mmol L^-1^ K_3_[Fe(CN)_6_]/K_4_[Fe(CN)_6_] + 0.1 mol L^-1^ KCl was recorded by the immunosensor after incubation in binary solutions [*Plasmodium vivax* lactate dehydrogenase antigen (Ag-*Pv*LDH) + interfering].

Selectivity studies were performed to assess whether the electrochemical immunosensor is specific for detecting the target analyte. The tests were carried out by incubating the device in binary solutions (Ag-*Pv*LDH + Glucose, Ag-*Pv*LDH + Glycine, and Ag-*Pv*LDH + Ag-*Pf*HRP2) for 1 h. Later, the electrochemical response was registered by the DPV technique under the same experimental conditions used in Ag-*Pv*LDH determination. The ∆Ipa was calculated from Ipa values before and after incubation in each binary solution. Fig. 6 is observed a low rebinding for all the molecules tested (glucose (5.31%), glycine (6.23%), and Ag-*Pf*HRP2 (7.76%)), suggesting that the immunosensor presents good selectivity for Ag-*Pv*LDH determination.

*In conclusion* - We presented the fabrication of an electrochemical immunosensor to determine Ag-*Pv*LDH, an important malaria biomarker. Cysteamine SAMs were successfully formed onto the GE surface, and then, Ab-*Pv*LDH were satisfactorily immobilised by covalent bonding. The device recorded a good performance in 10 to 50 µg mL^-1^ concentration range, with a LOD of 455 ng mL^-1^. The immunosensor showed a low ability to detect other molecules, with values less than 8% for the three interferents tested. Our immunosensor is simple, can be constructed in a short time, and present good sensitivity and selectivity for Ag-*Pv*LDH. Also, the application of egg yolk IgY antibodies in sensing platforms constitutes an attractive alternative for malaria diagnosis caused by *P. vivax*.
